# Exploring LSU and ITS rDNA Sequences for *Acanthamoeba* Identification and Phylogeny

**DOI:** 10.3390/microorganisms10091776

**Published:** 2022-09-03

**Authors:** Daniele Corsaro

**Affiliations:** CHLAREAS, 54500 Vandoeuvre-lès-Nancy, France; corsaro@gmx.fr

**Keywords:** *Acanthamoeba*, phylogenesis, LSU rDNA, ITS region, ITS-1, ITS-2

## Abstract

The identification and classification of strains of *Acanthamoeba*, a potentially pathogenic ubiquitous free-living amoeba, are largely based on the analysis of 18S rDNA sequences, currently delineating 23 genotypes, T1 to T23. In this study, the sequences of the ITS region, i.e., the 5.8S rDNA and the two internal transcribed spacers (ITS-1 and ITS-2), and those of the large subunit (LSU) rDNA of *Acanthamoeba* were recovered from amoeba genomes; the sequences are available in GenBank. The complete ITS–LSU sequences could be obtained for 15 strains belonging to 7 distinct lineages (T4A, T4D, T4F, T4G, T2, T5, and T18), and the site of the hidden break producing the 26Sα and 26Sβ was identified. For the other lines, either the LSU is partial (T2/T6, T7) or the ITS is fragmentary (T7, T10, T22). It is noteworthy that a number of sequences assigned to fungi turned out to actually be *Acanthamoeba*, only some of which could be affiliated with known genotypes. Analysis of the obtained sequences indicates that both ITS and LSU are promising for diagnostic and phylogenetic purposes.

## 1. Introduction

*Acanthamoeba* spp. (Amoebozoa, Discosea, Centramoebida) are ubiquitous free-living amoebae, abundant in a variety of natural and man-made environments; moreover, they are of medical interest because they can behave as opportunistic parasites for humans and other animals. Various species have been implicated in disseminated infections in multiple tissues and organs, with possible haematogenous spread to the central nervous system resulting in chronic granulomatous amoebic encephalitis (GAE); this is almost always fatal, especially in immunocompromised individuals [1,2]. *Acanthamoeba* is also a rare ocular pathogen causing infections normally confined to the cornea, leading to blinding amoebic keratitis (AK); nevertheless, other parts of the eye can also sometimes be invaded [3,4].

The *Acanthamoeba* life cycle includes active trophozoites and resistant double-walled cysts; they can be of three distinct morphologies, forming the basis of the traditional classification into groups 1–3 [5]. However, species identification and classification have been largely reassessed by nuclear small subunit (SSU) rDNA (18S rDNA) sequencing; delineating several genotypes corresponding to both classic and new species, and only partially consistent with the previous morphological classification. There are currently 23 genotypes (T1-T23), which in molecular phylogeny tend to cluster into four major lineages [6,7,8]. Morphological group 1 (MG1) species form the basal lineage; well separated from the remaining genotypes, with full correlation between morphological species and genotypes (T7-T9, T17, T18). The larger lineage includes: the T4 genotype (*A. castellanii* complex); divided into seven main groups, T4A to T4G, to which most environmental and clinical strains belong; the T3 and T11 genotypes (*A. griffini*/*A. hatchetti* group); and the T2/T6 clade (*A. palestinensis* group). The two other lineages are those of the *A. jacobsi*/*A.micheli* group (genotypes T15/T22 + T19/T13/T16), and of the *A. culbertsoni*/*A. healyi*/*A. bangkokensis* group (genotypes T10/T12/T14/T23 + T1/T20); while *A. lenticulata* (T5) and *A. pyriformis* (T21) form single lineages.

These relationships are largely consistent with those obtained using mitochondrial genes, such as SSU (16S) rDNA or the cytochrome c oxidase subunit I (Cox1) gene [6,9,10,11], as well as using sequences from the first intergenic transcribed spacer (ITS-1) of the nuclear rDNA [12]. However, the phylogenesis of *Acanthamoeba* is not fully resolved as some incongruences remain within and between the different trees; in addition, various genotype clusters are not well-supported. This is partly because, for all genotypes, genetic data are only available for 18S rDNA. Furthermore, it is likely that additional genotypes have yet to be discovered. In this study, the ITS region, i.e., the 5.8S rDNA flanked by ITS-1 and ITS-2, and the large subunit (LSU) of nuclear rDNA, were analysed; this was conducted in order to assess their possible use to improve phylogenetic resolution.

## 2. Materials and Methods

The *Acanthamoeba* genomes available on the NCBI portal were analysed to extract the ITS and LSU regions. The genomes were analysed by BLAST using as a query a sequence from the Neff strain (T4G genotype) spanning the complete rDNA operon (GenBank ID GU001160). Then, single or overlapping contigs with the region of interest were identified and the final sequences assembled. The different ITS-LSU sequences obtained were used for further genome screening and also, as a query to search by BLAST for closely related sequences in GenBank.

A first analysis was performed on LSU rDNA only, including other amoebae and fungi, to assess tree topology and confirm reliable identification of recovered sequences. Then, for sequences confirmed as *Acanthamoeba*, the ITS and LSU regions were aligned separately with that of *Balamuthia mandrillaris* (strain 2046) used as an outgroup. An 18S rDNA tree was constructed including the available sequences of the strains studied, to compare the results given by the different portions of the rDNA operon. Multiple alignments were performed using MAFFT and manually refined to exclude ambiguous sites using BIOEDIT. For alignment of the ITS region (ITS1-5.8S-ITS2), among the various programs tested (not shown), MAFFT with the L-INS-I option [13] was found to perform best. The multiple alignment thus obtained was visually checked to verify the correct positions of the 5.8S, as well as certain homologous parts identified in the ITS.

Molecular phylogenetic trees were built as previously described [14,15] with maximum likelihood (ML) (GTR *G* + I:4) using TREEFINDER [16], and neighbour-joining (NJ) (Kimura 2-P) and maximum parsimony (MP) using MEGA7 [17], with 1000 bootstraps. Pairwise similarity values for rDNA sequences were obtained with BIOEDIT by removing common and terminal gaps, and using all the sites and indels. Mean values within and between groups were then calculated manually.

## 3. Results and Discussion

### 3.1. Sequence Retrieval: General Features

The rDNA sequences were obtained from the available genomes of different *Acanthamoeba* strains; some were deposited under erroneous names, whose true identity was previously clarified by nuclear and mitochondrial SSU rDNA analysis [6]. Sequences of approximately 5400 bp were successfully extracted from fifteen of the twenty-four available genomes. Analysis of the remaining genomes gave incomplete or poor-quality results, even for 18S sequences. The obtained sequences covered the last 30 bp of 18S rDNA, the entire ITS region (about 1200 bp), and almost always the complete LSU rDNA sequence (near to 4300 bp); delineated by identifying the 3’end of the gene (GenBank ID L07635), as determined by Yang et al. [18]. The ITS region could not be completely recovered for *Acanthamoeba* sp. T22 because the corresponding contig contains various gaps, and it is very fragmented for *A. culbertsoni* (T10) and *A. astronyxis* (T7). Moreover, for *A. astronyxis*, only the 5’ end of the LSU (up to 2600 bp) could be detected. Another sequence considered here is that of strain BCP-EM3VG21-1 (hereafter BCP for simplicity) consisting of the complete 18S and ITS region, but only a short LSU (Table 1).

In *Acanthamoeba* as well as its close relative *Balamuthia*, ITS-1 and ITS-2 are long, exceeding the 250–300 nt size usually found in other protists [20]; as shown here (Table 1) for the two other analysed amoebae, the true *Rhizamoeba* [21] and *Vermamoeba* subtype 2 [19].

ITS-1 has a length between 309 and 512 nt; the shortest being found in *A. lenticulata* (T5) and the longest in *A. terricola* (T4G). Considering also the data of Köhsler et al. [12], some size distribution seems to occur. For example, the ITS-1 is 348–438 nt in T4A and T4B strains, and 440–480 nt in T4D strains; while it is 320–339 nt in the *A. palestinensis* group (T2/T6 line). A similar correlation between size and genotype is also found for ITS-2; this is always longer, varying between 480 nt for *A. lenticulata* (T5), 580–620 nt for T4A strains, around 640 nt for *A. mauritaniensis*, *A. rhysodes* (T4D) and *A. terricola* (T4G), and up to 700 nt for *A. triangularis* (T4F). The ITS-2 of *A. byersi* (T18) (Pb30/40 strain) is 1155 nt, and data for other MG1 species will be required to confirm if this is a feature of this lineage. The length variation of ITS-1 is largely due to multiple short repeats, mainly di- and tri-nucleotides (microsatellites) [12]. Microsatellite variations are also present in the ITS-2 of the different groups; although in all sequences, portions corresponding to ITS-2 helices could be identified (not shown). Similarity values for ITS-2 within groups are >75%, but drop to <60% even between related lines (Table 2).

The complete 5.8S rDNA sequence was found for all the strains analysed. Its length varies between 160 and 174 bp (Table 1), with 19 to 20 nucleotide substitutions (approx. 82% identity) between the MG1 species (T7 and T18) and all the others. The latter have much less variation, with only 0–6 nucleotide changes (96.3–100% identity). It is noteworthy that T7 and T18, although belonging to the same group, differ by 16 nt (91.9% identity). The sequence of the Neff strain used as a query (GenBank ID GU001160) has three mutations in 5.8S; these are not found in any other strain and are very likely the result of a sequencing error.

Complete LSU rDNA of 17 strains of six genotypes was obtained (Table 1). The sequences vary in length from approx. 4080 to 4350 bp, have no intron, and show similarity values close to those obtained from 18S rDNA (Table 3).

**Figure 1:**
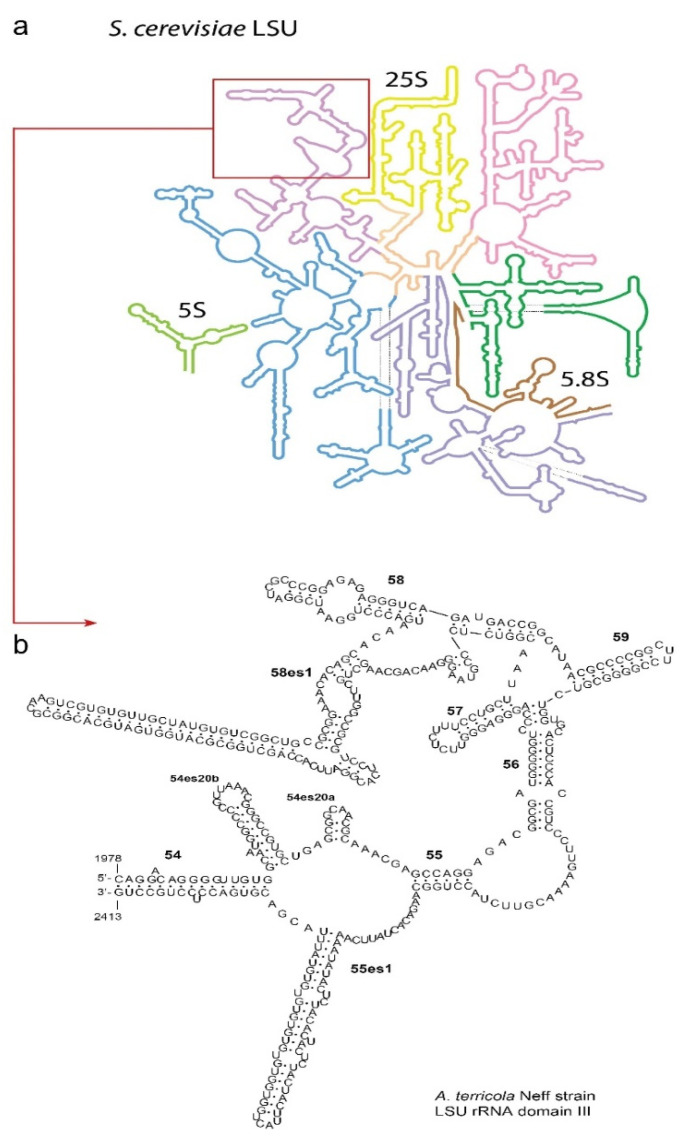
LSU structure: (**a**) an LSU rRNA secondary structure model of the yeast *Saccharomyces cerevisiae* (http://apollo.chemistry.gatech.edu/RibosomeGallery/, accessed on 24 July 2022). LSU domains I through VI are colour-coded, and 5S and 5.8S are also shown. The red rectangle indicates the region of interest here, in domain III, starting from stem 54; (**b**) the secondary structure of the above region of *Acanthamoeba terricola*, determined with Mfold. The AU-rich expansion segment 55es1 between 55 and 54 is the hidden break site.

Early works demonstrated that the LSU rRNA of *Acanthamoeba* (strain Neff) splits into two smaller, but unequal fragments: 26Sα and 26β, of approx. 2400 and 2000 nt, respectively; this is evidenced by gel electrophoresis of heat-denatured RNA and the formation of R-loops in the DNA–RNA hybridization assay [22,23]. The discontinuity in the LSU rDNA was located as a 200 bp gap on a restriction map of the cloned rDNA unit, between the Bgl II and Bam HI sites [23]. This region corresponds to domain III of the LSU rRNA, extending from stem 26’ to H62 (numbering after Petrov et al. [24]); in the *Acanthamoeba* sequences retrieved here, it exhibits two unusual expansion elements, forming however coherent structures in two-dimensional reconstructions (Figure 1).

One element is located inside the stem of H58 (58es1); the other in the loop between stems 55’ and 54’ (55es1); and their lengths vary between 50–130 and 10–80 nt among species, respectively. Interestingly, in all species, while the AT content of the whole LSU rDNA is 41.2–48.9%, it is very high for 55es1: between 63.2–86.3% (Table 4), which probably makes it unstable. The hidden break may therefore occur at this site, by the splicing of 55es1; thus, this produces the 26Sα and 26Sβ of about 2320 and 1880 nt, respectively. These results on several genotypes are entirely consistent with the previous ones based on the single Neff strain, and find new confirmation in the recent study by Natsidis et al. [25]; they analysed the hidden break in a larger number of eukaryotic LSU rRNA, retrieving for *Acanthamoeba* (Neff) almost identical prediction. The hidden break was not detected in the LSU of *Balamuthia* and the other amoebae analysed here.

### 3.2. Uncultured Fungi Turned Out to Be Acanthamoeba

**Figure 2:**
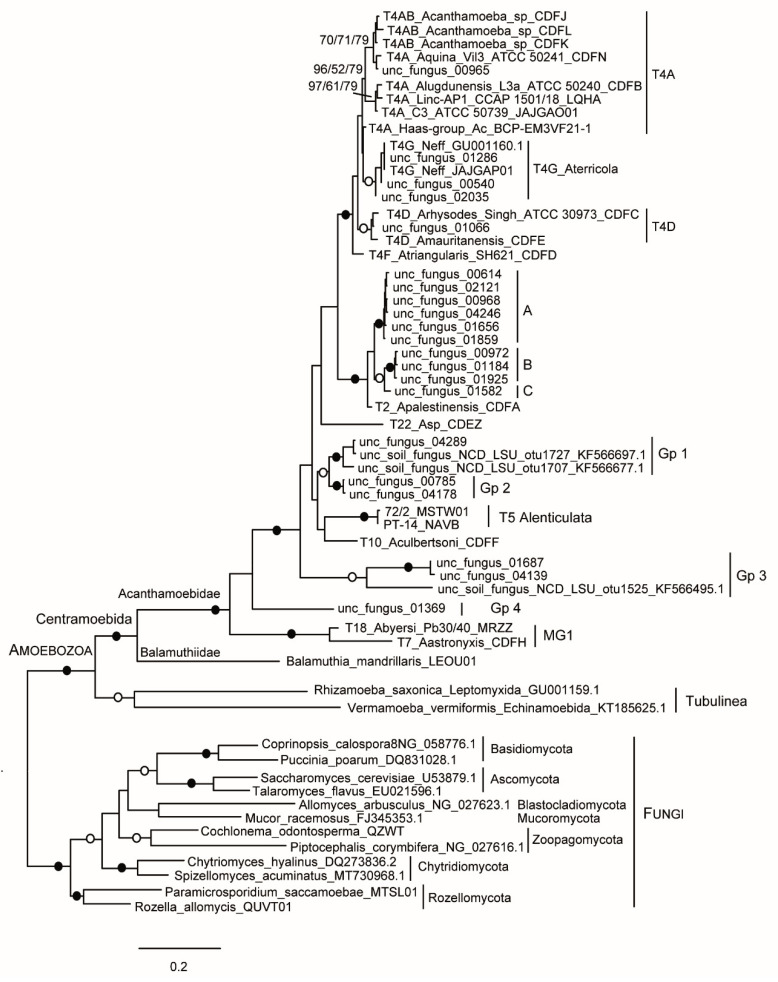
Molecular phylogeny based on LSU rDNA. The analysis includes complete LSU rDNA sequences for *Acanthamoeba* spp., the close relative *Balamuthia mandrillaris*; more distant amoebae belonging to the class Tubulinea; and main lineages of the fungal kingdom. For uncultured “fungal” clones, only partial sequences are available. At the nodes, there are bootstrap values (1000 replicates) for ML/NJ/MP, with filled and open circles for values 100 or >90% with all the methods.

BLAST search using *Acanthamoeba* ITS-LSU sequences retrieved from the analysed genomes yielded as close relatives 21 sequences of about 2600 bp (Kallberg et al., unpubl.); plus other shorter sequences of about 880 bp [26], all recovered from soil samples and deposited in GenBank as uncultured fungi. The longer sequences include both the complete ITS region and approx. 1300 bp of the LSU (domain I and part of domain II, up to stem 36); while the shorter sequences consist of the LSU alone (domain I up to H25a). Phylogenetic analysis based on the LSU sequences clearly indicates that these sequences are not fungi, but belong to *Acanthamoeba* (Figure 2). This misidentification is most likely due to the fact that in GenBank, ITS–LSU fungal sequences are overrepresented; while those of *Acanthamoeba* are absent, except for that of Neff strain.

Five clones emerge within *Acanthamoeba* T4, closely related to *A. quina* (T4A), *A. terricola* (T4G), or species of the T4D genotype. Ten other clones are clearly affiliated with *A. palestinensis* (T2), clustering in three groups, A to C; these could correspond to the other lineages of the T2/T6 clade. The remaining clones form three distinct lines, labelled groups 1/2, 3 and 4 for convenience; these are difficult to place due to the lack of available sequences for the other genotypes. The overall tree topology and mean within/between group similarity values for the LSU rDNA domains I/II (Table 5) are largely consistent with results typically obtained using 18S rDNA sequences.

Analysis of the ITS region of these clones, which are actually uncultured *Acanthamoeba*, also shows an interesting distribution by size and by group (Table 6); this is in agreement with the results presented above (Table 1) obtained from the genomes of *Acanthamoeba* strains.

### 3.3. ITS Phylogeny

The ITS region (ITS-1-5.8S-ITS-2) and ITS-1 alone from *Acanthamoeba* were used to assess the diagnostic potential and phylogenetic resolution of these portions. As only fragmentary portions for T7, T10, and T22 could be obtained, these genotypes were excluded from the ITS analysis; the analysis counts 38 complete sequences in total. Furthermore, 23 additional sequences are available for ITS-1; most were obtained from clinical or environmental samples (GenBank ID AF526424-AF526434; AY128512-AY128522) by Köhsler et al. [12]; and another, clone 20A, from a soil sample in Russia (GenBank ID MG706257; Oglodin et al., unpubl.).

**Figure 3:**
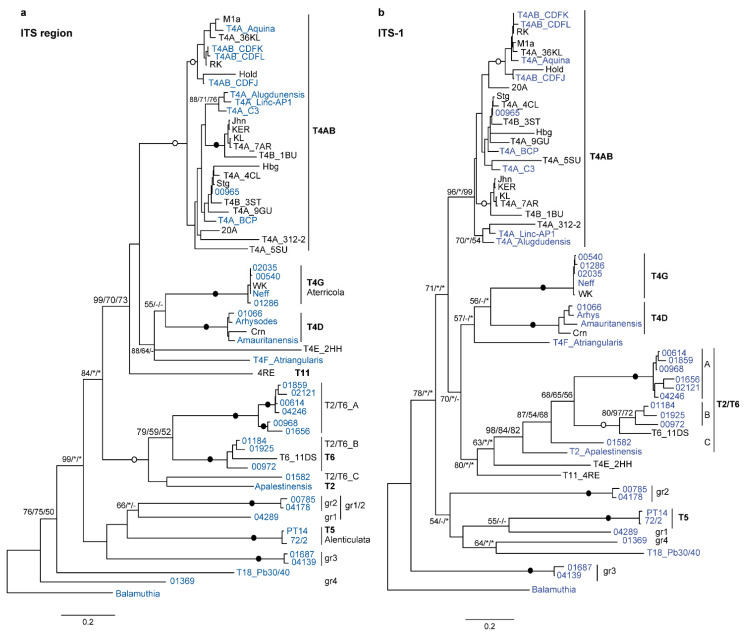
ITS phylogeny: (**a**) molecular phylogeny based on the entire ITS region; (**b**) molecular phylogeny based only on the ITS-1 sequence. The strains for which the entire ITS region is available are in blue (see Table 1 and Table 6). *Balamuthia mandrillaris* was used as an outgroup. At the nodes, bootstrap values (1000 replicates) for ML/NJ/MP are shown; filled and open circles, bootstrap support 100 or >90% with all the methods. *, node recovered but support <50%; -, node not recovered. ITS-1 could therefore be useful for identifying certain groups, but not for inferring phylogeny. In addition, the tree based on the ITS region is also in good agreement with that expected according to the 18S genotype. This is particularly evident for relationships between and within the closely related major groups, T4 (T4A to T4G) and the T2/T6 clade, down to the single strains; since for many of them, the 18S rDNA sequences are also available, allowing for an in-depth comparison of tree topologies (Figure 4).

The different lineages are all very well recovered by the phylogeny of the entire ITS region (Figure 3a), with a tree topology almost identical to that obtained with the LSU (Figure 2). Many subgroups are also well recovered using only ITS-1; however, producing inconsistent trees (Figure 3b).

It seems obvious that ITS-2 carries a stronger phylogenetic signal because the strains for which this portion is available cluster coherently with respect to the 18S topology. On the other hand, in ITS trees, the strains C3 and 9GU, for example, do not group together; however, they belong to the same branch (KA/E3 group). The same is true for strain 312-2 of *A. lugdunensis*, originally placed in an *A. quina*/*A. lugdunensis* complex (not “*A. quina-lugdunensis*”) [27], which comes out separated from L3a (the type strain of *A. lugdunensis*). This could be explained by the fact that for both 9GU and 312-2, only ITS-1 is available; allowing them to be placed in the main group (T4A), but not near the close relative.

Within the T2/T6 clade, it seems that group B can correspond to T6; it is sometimes incorrectly named “*A. operculata*” because a strain of *Acanthamoeba* was misdiagnosed as *Comandonia operculata* (actually synonymous of *Flamella*; for the correct naming of strains and species, see Corsaro [6]). By contrast, group A could be either lineage OX1 or lineage Page-45. In any case, the LSU and ITS data are congruent in supporting that the T2/T6 clade is composed of several distinct genotypes, as previously suggested [6,14].

There is a weak indication from preliminary data that group 1/2 could belong to the *A. jacobsi* or *A. culbertsoni* groups; however, representing neither T22 nor T10, the only members of both groups for which LSU sequences are available. Obviously, the ITS and LSU sequences of additional recognized strains will be required to elucidate their position. On the other hand, groups 3 and 4 remain unclassifiable; they could also correspond to new lines.

## 4. Conclusions

**Figure 4:**
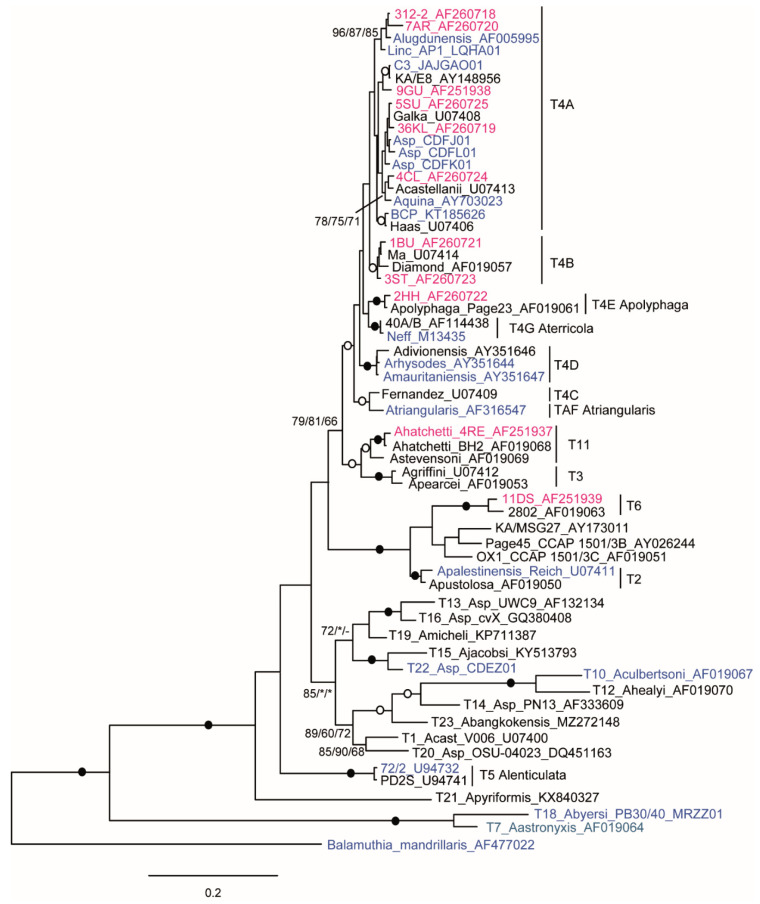
Molecular phylogeny of *Acanthamoeba* based on complete 18S rDNA. The strains for which ITS-1 alone or the entire rDNA operon is also available are shown in red and blue, respectively. The exceptions are T7, T10, and T22, and the BCP strain of T4A (see Table 1). The tree rooted on *Balamuthia mandrillaris*; bootstrap values (1000 replicates) for ML/NJ/MP are shown at the nodes; filled and open circles (100 or >90% support). *, node recovered but support <50%; -, node not recovered.

Phylogenetic analyses based on ITS and LSU largely support the results obtained by 18S. Separation within T4 into distinct groups, T4A to T4G, is always observed; except for some mixing between T4A and T4B, already reported for nuclear and mitochondrial SSU rDNA sequences [6,11]. The close affinity of the Linc-AP1 strain with *A. lungdunensis* (T4A) and not with *A. polyphaga* (T4E) [6] is confirmed. Moreover, the C3 strain turns out not to belong to *A. castellanii*, but to a distinct branch of T4A; this is evidenced by the 18S phylogeny (Figure 4). Similar results are obtained for the T2/T6 clade, for which the same lineages can be identified by the different portions of the rDNA operon. The LSU presents a variability comparable to that of 18S, or even slightly higher if the complete gene is considered (Table 3). Specific regions for the different genotypes and subgroups can already be identified in the partial LSU (domains I and II); which, for its small size (about 1300 bp), would be easier to sequence while ensuring useful data for the diagnosis and phylogenetics.

Various group I introns are present in the 18S of at least four genotypes (T3, T4, T5, and T15) [28,29]. However, they were not found in the LSU sequences analysed here; nonetheless, this does not exclude the possibility that other LSU may have introns.

ITS-1 was previously found to be tenfold more variable and correlated with 18S genotypes [12]; similar rate variability (Table 2) and genotype correlation (Figure 3) were found here for ITS-2, suggesting that these rDNA portions may be useful for the molecular identification of strains. Alignment of ITS-1 and ITS-2 is, however, difficult due to the large variations in length and sequence between the strains; this provides variable results depending on the program used. Including both 5.8S rDNA and additional sequences greatly improves the alignment, and produces a more reliable phylogenetic result. It can be expected that obtaining the ITS sequences of the remaining genotypes will make it possible to elucidate their secondary structure and to better identify the homologous regions to be retained in the multiple alignment.

ITS and partial LSU both clearly show their utility for *Acanthamoeba* sequence analysis; in addition, their use, separately or in combination, appears to better discriminate closely related strains. A major objective would obviously be to obtain the complete ITS and LSU sequences of at least one strain of each genotype. It would thus be possible to verify if the hidden break in the LSU occurs in all lineages, as well as to build a robust rDNA operon tree to better resolve the phylogeny.

## Figures and Tables

**Table 1:** Summary of the ITS and LSU sequence data.

Species	Strain	Culture Collection	SSU rDNA GT ^1^		Sequence Source	Length (bp)			
			Nuclear	mt		ITS1	ITS2	5.8S	LSU
*A. quina*	Vil3	ATCC 50241	T4A	T4a1	CDFN01	390	599	162	4259
*Acanthamoeba* sp.	undetermined	undetermined	T4AB	T4a1	CDFJ01	384	605	162	4272
*Acanthamoeba* sp.	undetermined	undetermined	T4AB	T4a1	CDFL01	348	621	162	4276
*Acanthamoeba* sp.	undetermined	undetermined	T4AB	T4a1	CDFK01	350	579	162	4272
*Acanthamoeba* sp.	BCP-EM3VF21-1	-	T4A	T4a3	KT185626	391	580	162	755 ^2^
*Acanthamoeba* sp.	C3	ATCC 50739	T4A	na	JAJGAO01	372	603	162	4264
*A. lugdunensis*	L3a	ATCC 50240	T4A	T4d	CDFB01	359	586	162	4251
*Acanthamoeba* sp.	Linc-AP1	CCAP 1501/18	T4A	T4d	LQHA01	387	594	162	4171
*A. mauritaniensis*	1652	ATCC 50253	T4D	T4e	CDFE01	480	641	162	4265
*A. rhysodes*	Singh	ATCC 30973	T4D	T4e	CDFC01	451	646	162	4279
*A. triangularis*	SH621	ATCC 50254	T4F	T4g	CDFD01	347	707	162	4284
*A. terricola*	Neff	ATCC 30010	T4G	T4f	JAJGAP01	512	637	162	4292
*A. palestinensis*	Reich	ATCC 30870	T2	T2	CDFA01	339	691	160	4174
*Acanthamoeba* sp.	unknown	undetermined	T22	T22	CDEZ01	>230 ^3^	>540 ^3^	160	4357
*A. culbertsoni*	Lilly-A1	ATCC 30171	T10	T10	CDFF01	na	na	160	4173
*A. lenticulata*	72/2	ATCC 50704	T5	T5	MSTW01	309	481	160	4091
*A. lenticulata*	PT14	-	T5 ^2^	T5	NAVB01	316	477	160	4082
*A. astronyxis*	undetermined	undetermined	T7	T7	CDFH01	na	na	172	2599 ^2^
*A. astronyxis*	undetermined	undetermined	T7	T7	CDFI01	na	na	172	2175 ^2^
*A. byersi*	Pb30/40	ATCC PRA-287	T18	T18	MRZZ01	346	1155	174	4103
*Balamuthia mandrillaris*	2046	-	-	-	LEOU01	396	603	154	3835
*Rhizamoeba saxonica*	161	CCAP 1570/2	-	-	GU001159	249	273	154	3677
*Vermamoeba vermiformis*	-	-	2 ^4^	-	KT185625	224	294	152	3768

^1^ Nuclear and mitochondrial (mt) SSU rDNA genotypes (GT), as defined in Corsaro [6]; ^2^ partial sequence; ^3^ presence of gaps; ^4^ as defined in Corsaro and Venditti [19].

**Table 2:** *Acanthamoeba* ITS-2 mean similarity values (%).

Group	*n*	T4AB	T4D	T4G	T4F	T2	T2/T6			Gp1	Gp2	Gp3	Gp4	T5
							A	B	C					
T4AB	9	**79.4–96.6**												
T4D	3	53.5	**89.9–92.6**											
T4G	4	56.0	57.2	**93.4–98.7**										
T4F	1	51.4	59.4	55.4	**100**									
T2	1	51.1	51.5	50.5	52.2	**100**								
T2T6 A	6	50.6	50.5	51.2	48.9	53.6	**76.7–95.1**							
T2T6 B	3	38.5	36.1	36.4	34.3	36.5	39.5	**77.7–95.9**						
T2T6 C	1	50.9	52.7	53.4	52.9	60.6	55.4	36.8	**100**					
Gp 1	1	52.0	50.8	52.6	50.7	51.1	51.7	38.4	49.0	**100**				
Gp 2	2	44.5	46.6	46.7	48.3	46.0	41.4	27.0	45.8	44.1	**87.1**			
Gp 3	2	50.5	53.0	51.3	52.3	51.9	49.1	34.6	51.1	51.4	49.7	**96.0**		
Gp 4	1	44.3	49.2	46.7	48.0	49.0	47.2	29.1	48.2	44.8	50.8	47.7	**100**	
T5	2	45.3	45.1	46.0	41.7	47.9	50.5	43.0	49.5	48.4	37.1	45.0	40.4	**96.3**
T18	1	34.3	38.8	36.7	38.2	38.0	34.9	21.2	38.5	34.0	43.1	38.8	44.3	31.9

For similarity within groups, the range of values is shown (bold). For the composition of the groups (*n*).

**Table 3:** Pairwise similarity values (%) for *Acanthamoeba* full-length 18S (top right, blue) and LSU (bottom left, green) rDNA sequences.

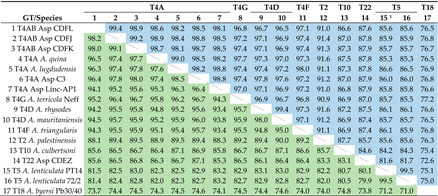

^1^ For 18S analysis, the sequence of PD2S was used because that of PT14 is incomplete.

**Table 4:** AT percentage values for *Acanthamoeba* LSU rDNA sequences.

GT	Species	Total LSU rDNA			55es1	
		Length (bp)	AT%		Length (bp)	AT%
T4AB	*Acanthamoeba* sp. CDFJ	4272	45.3		70	70.0
T4AB	*Acanthamoeba* sp. CDFL	4276	46.4		70	70.0
T4AB	*Acanthamoeba* sp. CDFK	4272	45.0		70	70.0
T4A	*A. quina*	4259	45.6		67	65.7
T4A	*Acanthamoeba* sp. C3	4264	44.6		60	65.0
T4A	*Acanthamoeba* sp. Linc-AP1	4171	45.0		54	64.8
T4A	*A. lugdunensis*	4251	45.0		53	64.2
T4D	*A. rhysodes*	4279	46.0		77	83.1
T4D	*A. mauritaniensis*	4265	45.9		65	76.9
T4F	*A. triangularis*	4284	45.3		74	77.0
T4G	*A. terricola*	4292	45.1		72	63.9
T2	*A. palestinensis*	4174	48.3		59	69.5
T10	*A. culberstoni*	4173	48.5		10	75.0
T22	*Acanthamoeba* sp.	4357	41.2		46	63.2
T5	*A. lenticulata* 72/2	4091	48.9		80	86.3
T5	*A. lenticulata* PT14	4082	48.7		74	85.1
T18	*A. byersi* Pb30/40	4103	46.0		44	68.2

**Table 5:** Mean pairwise similarity values (%) for the partial *Acanthamoeba* LSU rDNA sequences.

Group	*n*	T4					T2/T6				T22	Gp1/2		T10	T5	Gp3	Gp4	MG1
		T4A	T4G	T4D	T4F		T2	A	B	C		Gp1	Gp2					
T4A	8	**98.4**																
T4G	4	98.0	**99.9**															
T4D	3	96.6	97.0	**98.4**														
T4F	1	96.7	97.3	96.1	**100**													
T2	1	87.7	88.4	88.6	89.0		**100**											
T2T6 A	6	87.9	89.2	88.7	88.6		95.2	**99.1**										
T2T6 B	3	86.0	87.2	86.7	87.4		94.0	93.6	**98.4**									
T2T6 C	1	88.0	88.7	88.2	88.7		95.4	95.6	95.3	**100**								
T22	1	85.8	85.7	85.4	85.5		80.5	80.9	79.5	80.8	**100**							
Gp 1	1	87.0	87.7	86.7	87.8		84.5	85.1	82.9	85.0	85.2	**100**						
Gp 2	2	88.0	88.4	88.3	88.7		84.5	84.4	84.0	84.4	84.4	90.6	**99.2**					
T10	1	86.1	86.8	86.2	86.4		84.1	84.7	84.2	84.7	82.6	87.5	86.6	**100**				
T5	2	80.1	80.4	80.2	80.0		80.6	81.6	80.7	81.0	76.1	78.9	78.2	78.8	**99.6**			
Gp 3	2	75.2	75.5	75.3	75.4		75.1	74.9	73.9	74.3	75.1	75.2	74.9	74.6	75.0	**99.1**		
Gp 4	1	74.8	75.6	75.2	75.6		74.6	74.0	73.7	74.2	73.2	75.2	75.2	74.0	69.9	69.3	**100**	
MG1	2	71.8	72.3	71.7	72.6		71.2	70.6	70.2	71.3	71.3	71.4	71.2	71.7	68.8	70.5	71.5	**84.2**

Values within each group are in bold.

**Table 6:** ITS lengths of the uncultured *Acanthamoeba*.

Group		Clone	GenBank ID	Length (bp)			*Acanthamoeba* Line
				ITS1	ITS2	5.8S	
T4	T4A	00965	OU939676	427	580	162	T4A
	T4G	02035	OU940742	514	651	162	T4G
		01286	OU939992	520	656	162	
		00540	OU939249	512	640	162	
	T4D	01066	OU939772	444	653	162	T4D
T2/T6	A	00614	OU939321	325	590	162	T2/T6
		01859	OU940566	323	580	162	
		00968	OU939674	322	573	162	
		04246	OU942952	325	589	162	
		02121	OU940828	311	587	162	
		01656	OU940362	315	586	162	
	B	01184	OU939890	381	296	164	T6
		01925	OU940630	405	291	164	
		00972	OU939679	387	305	164	
	C	01582	OU940289	297	675	163	T2/T6
Gp1/2	1	04289	OU942995	325	566	160	undetermined
	2	00785	OU939503	483	865	160	
		04178	OU942885	482	841	159	
Gp3	3	01687	OU940393	275	650	155	undetermined
		04139	OU942845	277	660	155	
Gp4	4	01369	OU940075	300	768	163	undetermined

## Data Availability

Part of the data used in this study was obtained from publicly available genomes through computational work; therefore, it is not suitable for GenBank submission. Refined sequences are available from the author upon reasonable request.
